# Exercise improves endothelial progenitor cell’s function in mice with Type 2 diabetes via gut microbiota modulation

**DOI:** 10.3389/fcimb.2025.1606652

**Published:** 2025-08-28

**Authors:** Xia Dai, Haiyan Chen, Milei Zhang, Qiong Yang, Zheng Huang, LiAn Tang

**Affiliations:** ^1^ Department of Endocrinology, The First Affiliated Hospital of Guangxi Medical University, Nanning, China; ^2^ Anesthesia and Operation Center, The First Affiliated Hospital of Guangxi Medical University, Nanning, China; ^3^ Department of Endocrinology, Baise People’s Hospital, Baise, China; ^4^ Department of Gastrointestinal Oncology, Affiliated Tumor Hospital of Guangxi Medical University, Nanning, China; ^5^ Department of Respiratory and Critical Care Medicine, The First Affiliated Hospital of Guangxi Medical University, Nanning, China

**Keywords:** exercise, diabetes, fecal microbiota transplantation, endothelial progenitor cells, GLP-1

## Abstract

**Introduction:**

Evidence has proved that exercise increases migration and tube formation of rat EPCs. But the mechanism behind the improved function of EPCs by exercise remains unclear.

**Methods:**

This study conducted 8-week exercise interventions (aerobic, resistance, or combined) in 6-week-old type 2 diabetic mice, assessing post-exercise glucose, weight, GLP-1, and gut microbiota. Mice with optimal outcomes were selected as fecal donors for microbiota transplantation via gavage. Recipient mice were evaluated for GLP-1, microbiota changes, and endothelial progenitor cell (EPC) proliferation/migration.

**Results:**

Exercise altered microbial composition (e.g., increased Prevotellaceae and Ligilactobacillus), while fecal microbiota transplantation(FMT) enriched Akkermansia. Notably, FMT elevated plasma Glucagon-like peptide-1 (GLP-1) levels by 0.92 pmmol/L (P < 0.001) compared to controls, surpassing the modest, non-significant effects of exercise alone. Critically, FMT enhanced EPC’s proliferation (P < 0.007 vs. controls) and migration (P < 0.05), mirroring exercise-induced improvements. While exercise reduced body weight (e.g., 10.58 g in aerobic training (AT), P < 0.001) and blood glucose, FMT amplified these metabolic benefits, lowering glucose by 9.22 mmol/L (P < 0.001).

**Discussion:**

Our findings suggest that exercise improves EPC’s function in diabetic mice via gut microbiota modulation, with FMT synergistically enhancing GLP-1 secretion. The identified microbiota (Prevotellaceae, Ligilactobacillus, Akkermansia) may serve as therapeutic targets for T2DM(T2DM) and its cardiovascular complications.

## 1 Background

An estimated 536.6 million people had diabetes mellitus (DM) as of 2021, alarmingly, the prevalence of DM is still increasing, with 783.2 million people projected to be diagnosed with DM by 2045 (Sun et al., 2022). diabetic cardiovascular disease has a similar prevalence, impacting 32% of diabetics worldwide (Einarson et al., 2018a). Likewise, the addition of CVD increases the cost of diabetes care by $3500-$10000 (Einarson et al., 2018b). Endothelial progenitor cells (EPCs) are a population of bone marrow-derived cells that can facilitate endothelial repair and reverse dysfunction. Hyperglycemia injures EPCs, therefore, contribute to the development of diabetic cardiovascular disease (Raleigh et al., 2024).

Evidence has proved that exercise increases migration and tube formation of rat EPCs (Li et al., 2024). Our research team also showed that aerobic exercise, resistance exercise, and aerobic combined resistance exercise improved proliferation, migration, adhesion, and angiogenesis in diabetic mice (Dai et al., 2020; Zhai et al., 2020). But the mechanism behind the improved function of EPCs by exercise remains unclear.

Studies has revealed that metabolites of intestinal flora induces endothelial cell dysfunction and suppression of EPCs production in circulation (Ma et al., 2017; Chou et al., 2019). In order to verify the relationship between exercise, intestinal flora and EPCs, this study conducted exercise intervention, intestinal flora sequencing, fecal microbiota transplantation and other interventions in diabetic mice. The changes of intestinal flora after intervention were detected, so as to reveal the related flora to improve EPCs.

## 2 Materials and methods

### 2.1 Study design

Ethical approval for the study was obtained from the Institutional Animal Care and Use Committee at Guangxi Medical University. Male db/db mice with T2DM, aged eight weeks, were procured from the Nanjing Biomedical Research Institute of Nanjing University, located in Nanjing, China.

The db/db mice were characterized by the presence of mutant alleles of the leptin receptor gene, resulting in the manifestation of an obese phenotype accompanied by T2DM by the age of six weeks. The mice were housed in conditions of a 12:12-hour light-dark cycle, with 4–5 animals per cage allocated to the same treatment group. They were provided with standard chow for sustenance.

Animals demonstrating a recurring blood glucose concentration exceeding 11.1 mmol/l were subject to random assignment into the control, combined aerobic and resistance training(AT+RT), AT and resistance training(RT) group. The mice assigned to the AT, RT, and AT+RT groups underwent 8 weeks of corresponding training regimens, with specific exercise protocols detailed in our previous publication (Dai et al., 2020).

### 2.2 Exercise training

The mice were stratified into three exercise intervention groups to identify the most effective exercise modality for enhancing gut microbiota profiles. The optimal group would subsequently serve as fecal microbiota transplantation (FMT) donors. Control group mice received no structured exercise intervention, whereas the exercise intervention groups underwent either aerobic training (AT), resistance training (RT), or a combination of both (AT+RT).

Mice in the AT+RT group followed an alternating-day exercise schedule: aerobic training (AT) was conducted on Monday, Wednesday, and Friday, while resistance training (RT) was performed on Tuesday, Thursday, and Saturday. The control group mice remained sedentary throughout the study period without participating in any structured exercise regimen.

#### 2.2.1 AT protocol

The AT group mice underwent an 8-week progressive treadmill training program using a specialized rodent treadmill (WDW-1, Beijing North Rui Future Analytical Instrument Co., Ltd.). The protocol began with a 1-week acclimation phase at 10 m/min for 10 minutes per day (6 days/week), followed by 7 weeks of gradually intensified training where the running speed was progressively increased from 13 to 17 m/min for 50 minutes per day while maintaining the 6 days/week training frequency.

#### 2.2.2 RT protocol

A custom-designed 1-meter ladder with 2-cm grid spacing was positioned at an 85° incline. Mice performed ladder climbing exercises with progressively increasing loads: initial resistance was set at 10% of body weight attached to the tail, gradually increasing to 70% over the 8-week training period. Training sessions consisted of 3 sets × 5 repetitions (with 1-minute inter-repetition and 2-minute inter-set rest periods), conducted 6 days/week. Manual stimulation (gentle tactile cues) was provided to maintain climbing motivation until task completion.

### 2.3 Antibiotic administration and microbiota transplantation

Mice in the FMT and NS group were continuously intragastrically administrated 200μL broad-spectrum antibiotic mixture containing ampicillin (0.5 mg/mL), vancomycin (0.25 mg/mL), polymyxin B (1mg/mL), and metronidazole (0.5 mg/mL) (antibiotic cocktail, ABX) for 4 weeks. After antibiotic treatment, 16S rRNA was measured in the feces to ensure that the effects of antibiotics on the microbiota were similar. 0.5 ml cecal/colon supernatant (500mg/mL) from mice in the AT+RT group were intragastrically administered to mice in the FMT group for 14 consecutive days, and the feces of each group after transplantation were collected. In addition to the above-mentioned mice, mice in the NS group were intragastrically administered 0.9% normal saline instead of the donor supernatant.

### 2.4 DNA extraction and microbial diversity analysis

Colon contents and feces were stored in liquid nitrogen for genomic DNA extraction. After a quickreturn to normal temperature in a 25 C water bath, genomic DNA from the colon contents and feces were extracted using the EZNA Stool DNA Kit (Omega Bio-tec, Norcross, GA, USA), and the extraction quality was observed by 1% agarose gel electrophoresis (AGE). One part of the extracted DNA was amplified by PCR using primers 341F (5’-CCTAYGGGRBGCASCAG-3’ and 806R (5’-GGACTACNNGGGTATCTAAT-3’) belonging to the V3-V4 variable region of 16S rDNA. Afterwards, the PCR products were examined by 2% AGE and recycled by gel-cutting using an AxyPrepDNA Gel Recovery Kit (Axygen, Union, CA, USA) for 16S rDNA sequencing (Illumina, San Diego, CA, USA).

### 2.5 16S rRNA amplification and sequencing and biosignal analysis

Bacterial DNA was extracted from colon contents and feces, purified, quantified, and sequenced using the Illumina MiSeq platform. Sequencing libraries were prepared using the TruSeq Nano DNA LT Library Prep Kit (Illumina). The aforementioned sequences were merged by 97% sequence similarity and partitioned by Operational Taxonomic Units (OTU), with QIIME software and UCLUST, a sequence alignment tool. The obtained abundance matrix was used to calculate α-diversity. According to the results of OTU classification and taxonomic status identification, the specific composition of each sample at each taxonomic level can be obtained. Using Mothur, QIIME, and R-software, principal component analysis(PCA) allows presentation of difference in multiple data in a two-dimensional scatter plot, whose distance between two dots(samples) indicates similarity between two samples in feature composition. The 16S rRNA gene sequence was predicted in KEGG Pathway Database (KEGG) and Cluster of Orthologous Groups of Proteins (COG), which are two functional spectrum databases, using Phylogenetic Investigation of Communities by Reconstruction of Unobserved States (PICRUSt). Annotation information corresponding to each functional spectrum database was obtained for each sample, and the abundance matrix of predicted functional groups was obtained. A Venn diagram was created. The shared/unique OTU between samples and groups was visualized with a “Venn Diagram” that was created with R software.

### 2.6 Cell experiment and analysis with plasma samples

Methods to isolate, culture andidentify bone marrow-derived EPCs have been described in detail in a previously published article. GLP-1 was detected by ELISA according to the instruction of ELISA kit.

#### 2.6.1 EPCs proliferation assay

Suspended EPCs were seeded onto collagen-coated 96-well plates at a density of 3.6–4.0 × 10³ cells/well and cultured for 24 h. After incubation, 10 µL of CCK-8 solution (Dojindo Molecular Technologies, Inc.) was added to each well, followed by an additional 4 h of incubation under light-protected conditions. Prior to optical density (OD) measurement, the plate was gently agitated for 10 sec to ensure uniform distribution. OD values were recorded at 450 nm using a microplate reader at 0 h, 24 h, 36 h, and 72 h post-CCK-8 addition.

#### 2.6.2 EPC migration assay

A straight reference line was drawn on the back of a 6-well plate using a marker. Then, 1–3 × 10^6^ EPCs were seeded per well and cultured for 24 h. After incubation, the plate was washed three times with 1× PBS buffer. The cells were maintained in serum-free medium at 37°C under 5% CO_2_ (Thermo Fisher Scientific, Inc.). Cell migration was assessed by capturing images at 0 h and 48 h using an inverted microscope (100× magnification) and analyzing them with ImageJ software (National Institutes of Health).

### 2.7 Data and statistics

The data for the physiological characteristics of the rats were expressed as the mean ± standard deviation (SD). Statistical analysis of differences between the different groups was performed with two-way ANOVA and then tested using Tukey’s true significant differences test. When only two groups were compared, Students’ t-test was used. All analyses were performed using Prism 8.0 (GraphPad, La Jolla, CA, USA) software.

## 3 Results

### 3.1 Identification of intestinal microbiome

**Figure 1:**
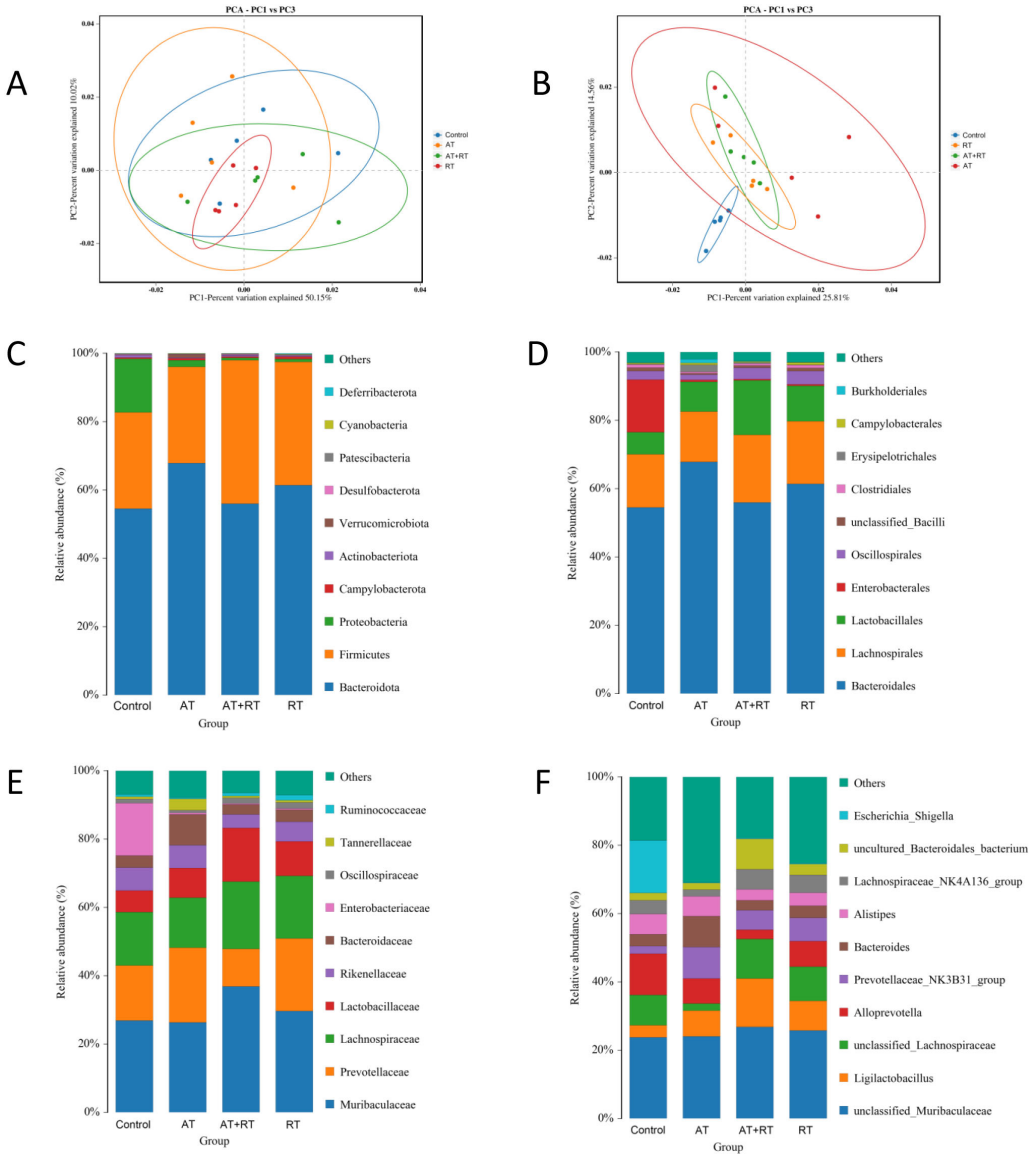
Composition of the intestinal microbiome in db/db mice after exercise intervention. **(A, B)** The relative abundances of 16S rDNA and metagenome at each genus level were used in Principal Component Analysis (PCA). X- and Y-axes represent the1st and 2nd components of the PCA plot, respectively. **(C–F)** Community accumulation histogram showing the relevant abundance of top 10 phyla, order, family and genera detected by 16S rDNA sequencing. AT, aerobic training; RT, resistance training, AT+RT: combined aerobic and resistance training.

To analyze the differences in intestinal microbiome between the control, AT, RT and AT+RT groups globally, a biplot of the generic-level PCA was created, as represented in **Figure 1**. PC1 and PC2 showed approximately 50% variability before and 25% after exercise. Regarding the confidence intervals between the AT, RT and AT+RT(exercise group), there was only a small overlap. However, the microbes of between control and exercise group(AT, RT and AT+RT)were different and further explored the data. At the genus level, Prevotellaceae NK3B3l group and Ligilactobacillus were more numerous in AT, RT and AT+RT then control group.

### 3.2 The genus levels of microbes in db/db mice

#### 3.2.1 Difference of microbes after exercise

Analysis results indicate that there were dozens of significant variations at the genus level (p < 0.05) between groups. Compared with db/db mice of the control group, Escherichia_Shigella and Rikenella were found to be downregulated in AT and RT, while Prevotellaceae_UCG_001 and Faecalibaculum in AT and Prevotellaceae_UCG_001 and Ruminococcus in RT were upregulated. Escherichia Shigella, Bacillus, [Eubacterium]xylanophilum group and [Eubacterium] nodatum _group were less enriched in AT+RT then the control group. Uncultured Bacteroidales bacterium, unclassified Rhodospirillales, Ligilactobacillus, uncultured Clostridiales bacterium and unclassified Erysipelotrichaceae were more abundance in AT+RT then the control group.

#### 3.2.2 Difference of microbes after fecal microbiota transplantation

**Figure 2:**
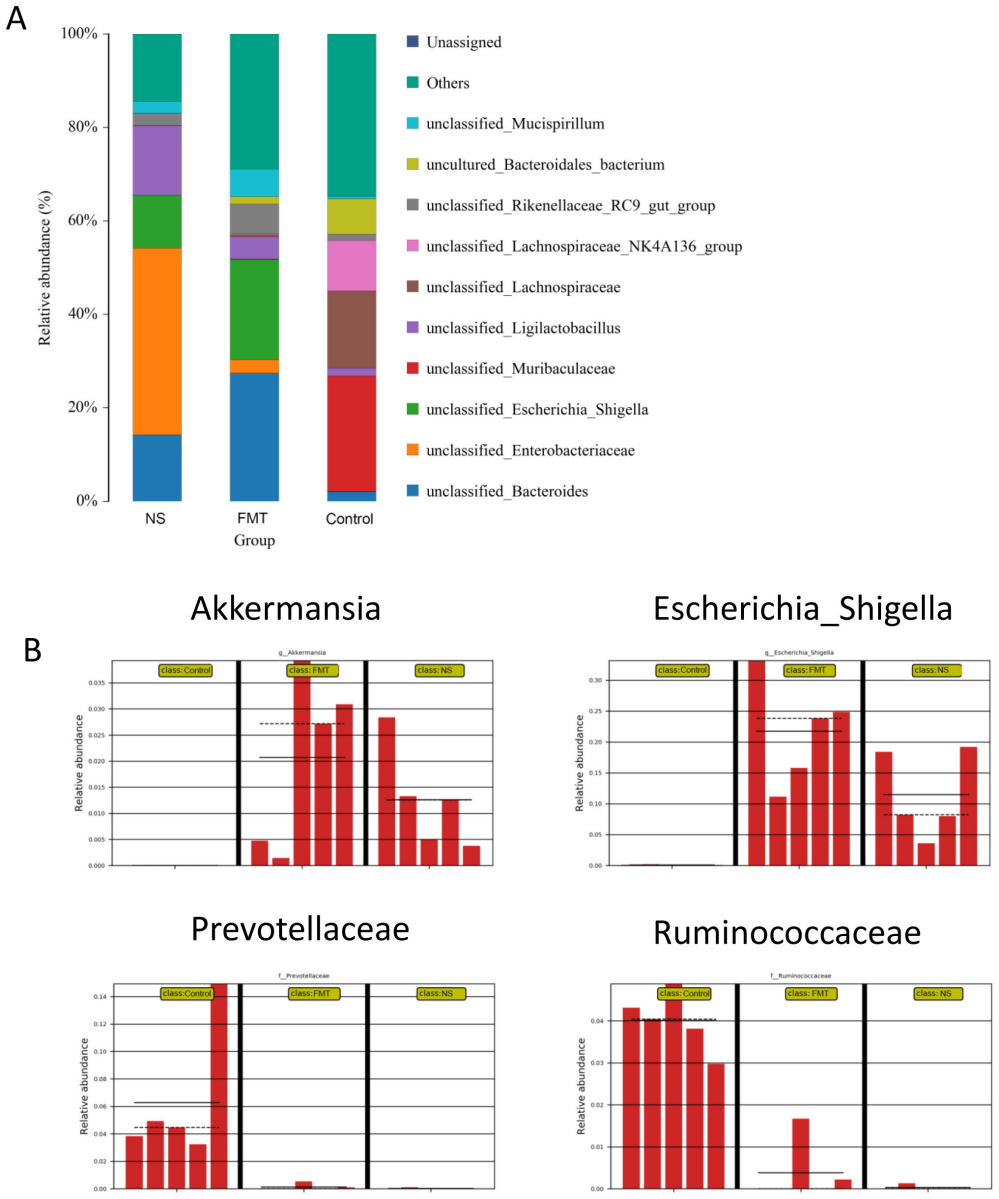
Composition of the intestinal microbiome in db/db mice after fecal microbiota transplantation. **(A)** Community accumulation histogram showing the relevant abundance of top 10 species detected by 16S rDNA sequencing, **(B)** fecal microbiota with significant differences between the NS, FMT and control groups. * p < 0.05. FMT, fecal microbiota transplantation; NS, normal saline.

**Figure 2** showed that there were significant differences in intestinal prokaryotes in db/db mice after fecal microbiota transplantation. Akkermansia was upregulated in mice of the FMT group, which have received fecal microbiota transplantation from mice trained with 8-weeks combined aerobic and resistance exercise for 2 weeks. However, upregulation of Escherichia_Shigella and downregulation of Prevotellaceae and Ruminococcaceae in the FMT and NS group were along with the benefit, which maybe the consequence of antibiotic therapy before fecal microbiota transplantation.

### 3.3 Intergroup differences in functional annotations

#### 3.3.1 Differences in functional annotations after exercise

**Figure 3:**
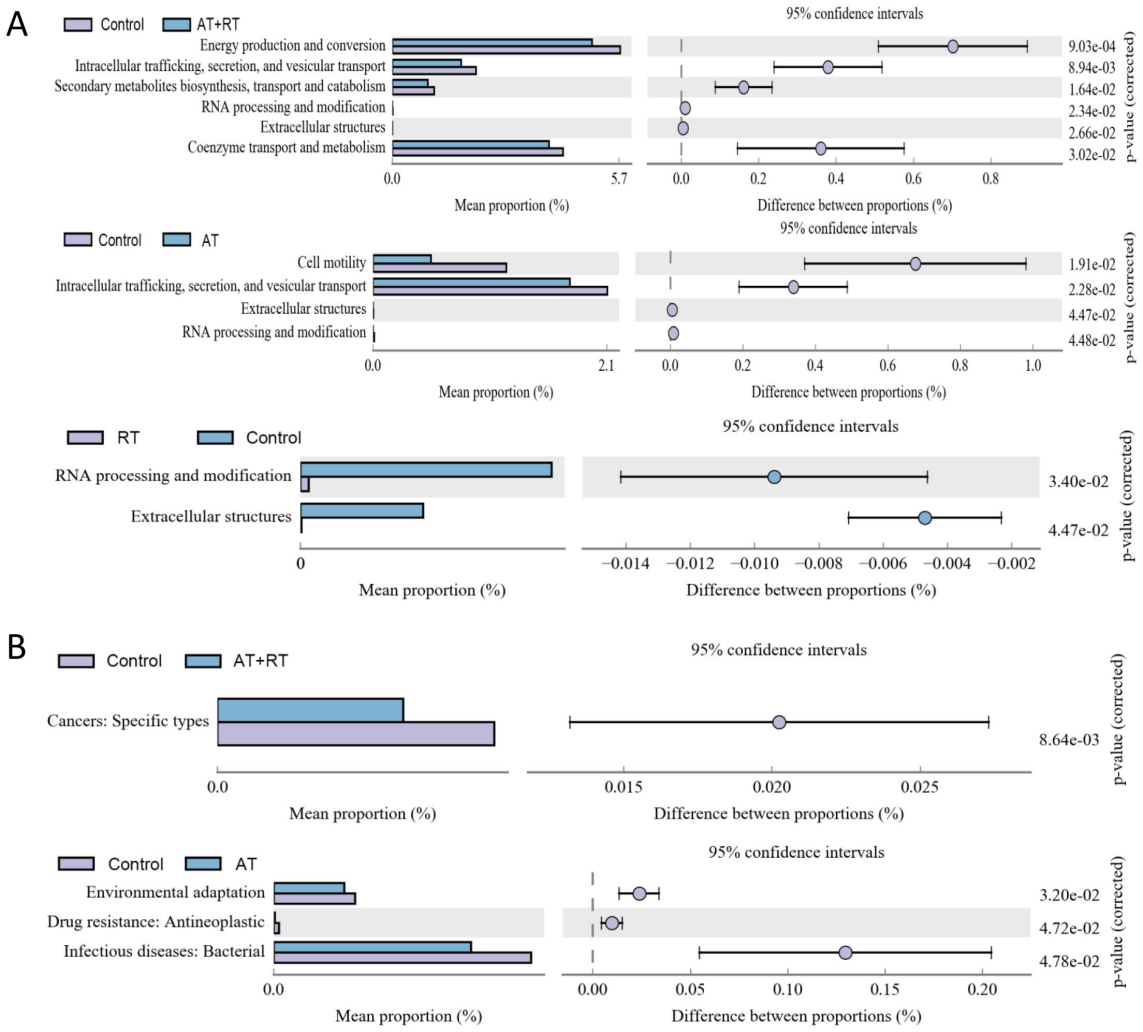
The annotation results from COG and KEGG database after exercise intervention. **(A)** the COG annotation results with significance, **(B)** the level 2 sequence annotation results from the KEGG database. AT, aerobic training; RT, resistance training; AT+RT, combined aerobic and resistance training.

Significantly different sequences obtained by COG annotation are shown in **Figure 3**. compared with the control group, Energy production and conversion: Intracellular trafficking, secretion, and vesicular transport: Secondary metabolites biosynthesis, transport and catabolism: Coenzyme transport and metabolism were downregulated in the AT+RT group, Cell motility: Intracellular trafficking, secretion, and vesicular transport were decreased in the AT group and RNA processing and modification: Extracellular structures were reduced in the RT group. Meanwhile, Compared with the control group, Cancers: Specific types declined in the and Environmental adaptation, Drug resistance: Antineoplastic, Infectious diseases: Bacterial were descended in the AT group.

#### 3.3.2 Differences in functional annotations after fecal microbiota transplantation

**Figure 4:**
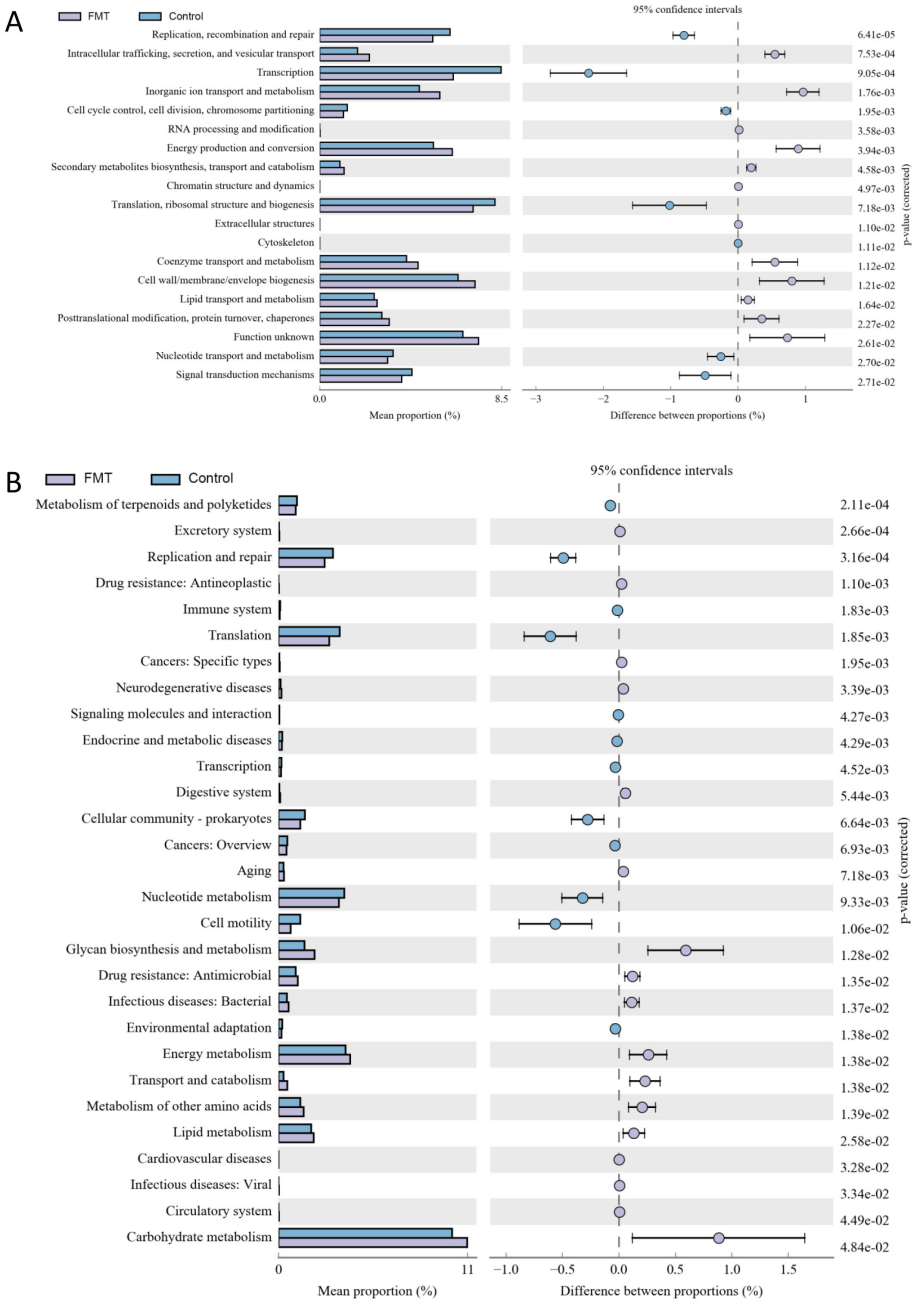
The annotation results from COG and KEGG after fecal microbiota transplantation. **(A)** the COG annotation results with significance, **(B)** the level 2 sequence annotation results from the KEGG database. FMT, fecal microbiota transplantation; NS, normal saline.

In **Figure 4**, Energy production, conversion and metabolism were more active in the FMT group then the control group. On the contrary, Replication, repair, Transcription and Signal transduction mechanisms were more positive in the control group then the FMT group. The level 2 KEGG pathway annotation difference showed that Energy metabolism of lipid, amino acids and carbohydrate had increased in the FMT group and Replication, Translation, Cell motility and Nucleotide metabolism had upregulated in the control group.

### 3.4 Intergroup differences in body weight, blood glucose, GLP-1, cell function

#### 3.4.1 Differences in blood glucose

**Figure 5:**
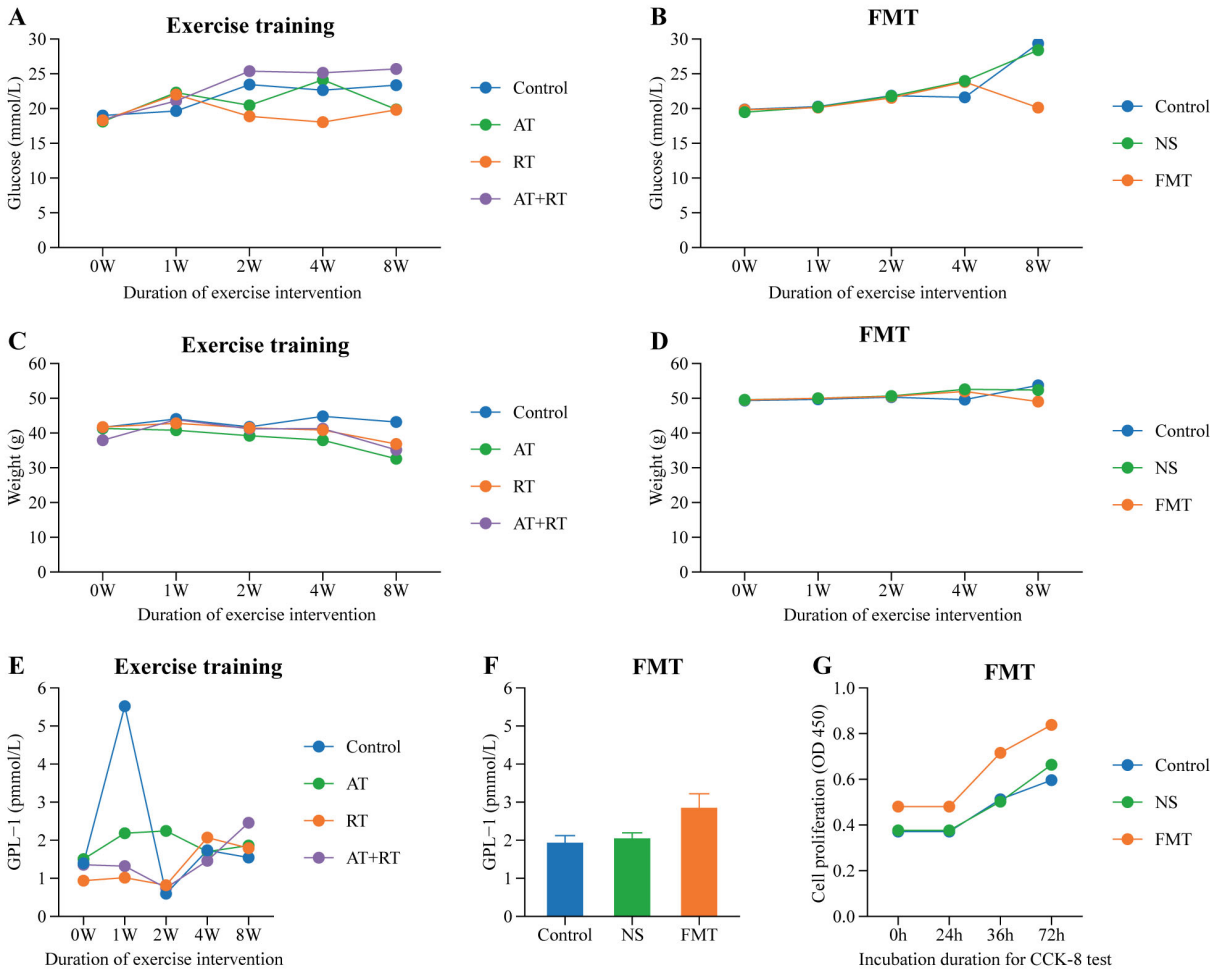
Difference of glucose, weight, GLP-1 and EPC’s proliferation among group. **(A)** Difference of glucose in mice with exercise intervention, **(B)** Difference of glucose in mice with fecal microbiota transplantation, **(C)** Difference of weight in mice with exercise intervention, **(D)** Difference of weight in mice with fecal microbiota transplantation, **(E)** Difference of GLP-1 in mice with exercise intervention, **(F)** Difference of GLP-1 in mice with fecal microbiota transplantation, **(G)** Difference of EPC’s proliferation in mice with fecal microbiota transplantation. AT, aerobic training; RT, resistance training; AT+RT, combined aerobic and resistance training; FMT, fecal microbiota transplantation; NS, normal saline.

With prolonged intervention, AT, RT, and AT+RT all significantly increased blood glucose levels in type 2 diabetic mice, though the differences were not statistically significant. However, in the FMT group, which received fecal microbiota transplantation from exercise-intervened mice, the blood glucose levels of type 2 diabetic mice were 9.22 mmol/L lower than those in the NS group (which received saline) (P < 0.001) and 8.27 mmol/L lower than those in the control group (P < 0.001). See more details in **Figures 5A, B**.

#### 3.4.2 Differences in body weight

As clearly shown in **Figures 5C, D**, the body weight of type 2 diabetic mice in the AT, RT, and AT+RT groups decreased with prolonged exercise intervention, with the most significant reduction observed in the AT group. The AT group exhibited a 10.58 g decrease in body weight compared to the control group (P < 0.001). Similarly, mice in the FMT group, which received fecal microbiota transplantation from exercised mice, also showed a significant reduction in body weight, being 4.69 g lower than the control group (P < 0.001).

#### 3.4.3 Differences in GLP-1

All three exercise modalities slightly increased plasma GLP-1 levels in type 2 diabetic mice, though the differences were not statistically significant. In contrast, the FMT group (receiving fecal microbiota transplantation) exhibited significantly higher plasma GLP-1 levels—0.92 pmol/L higher than the control group (P < 0.001) and 0.80 pmol/L higher than the NS group (P < 0.001). These findings indicate that exercise does not directly elevate plasma GLP-1 in type 2 diabetic mice; rather, the gut microbiota serves as the key regulator of plasma GLP-1 levels. See more details in **Figures 5E, F**.

#### 3.4.4 Intergroup differences in EPC’s cell function

**Figure 6:**
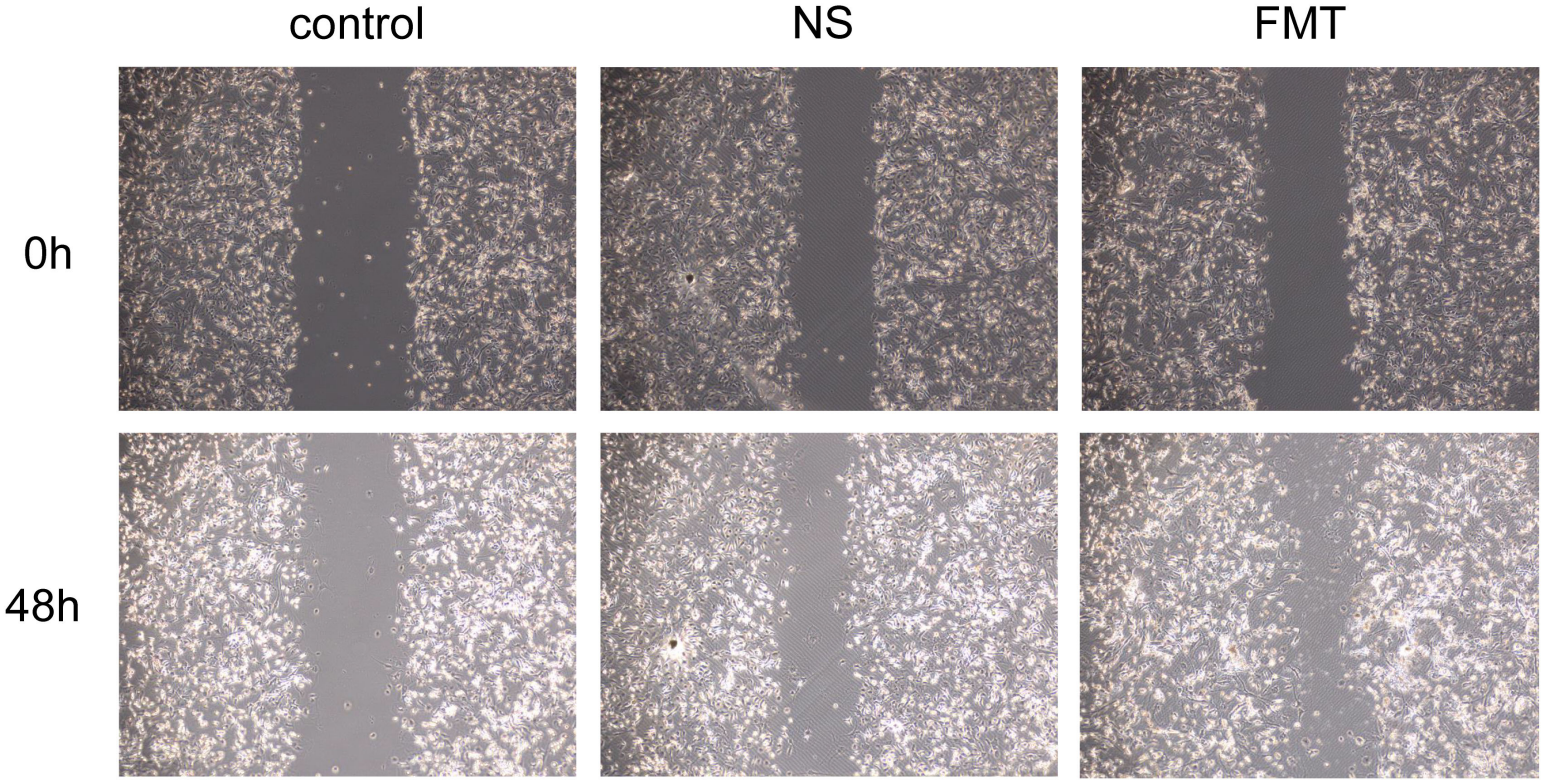
Differences in EPC’s migration after fecal microbiota transplantation. FMT, fecal microbiota transplantation; NS, normal saline.

As shown in **Figure 5G**, the EPC proliferation capacity in type 2 diabetic mice of the fecal microbiota transplantation (FMT) group was significantly stronger than that in the control group (P < 0.007) and the NS group (P < 0.031). **Figure 6** demonstrates that compared to both the control and NS groups, the FMT group exhibited significantly enhanced EPC migration capability in type 2 diabetic mice (P < 0.05).

## 4 Discussion

The present study elucidates the dynamic interplay between exercise interventions, FMT, and functional shifts in the intestinal microbiome of db/db mice, a model of metabolic dysfunction. Our findings highlight that Escherichia_Shigella and Rikenella were found to be downregulated in AT and RT, while Prevotellaceae_UCG_001 and Faecalibaculum in AT and Prevotellaceae_UCG_001 and Ruminococcus in RT were upregulated. Notably, FMT elevated plasma GLP-1 levels compared to controls, surpassing the modest, non-significant effects of exercise alone. Critically, FMT enhanced EPC proliferation and migration, mirroring exercise-induced improvements. While exercise reduced body weight and blood glucose, FMT amplified these metabolic benefits.

### 4.1 Exercise-mediated improvement of endothelial progenitor cell function in T2DM mice is gut microbiota-dependent

The most compelling result observed in this study is that FMT elevated plasma GLP-1 levels compared to controls, surpassing the modest, non-significant effects of exercise alone. Critically, FMT enhanced EPC proliferation and migration, mirroring exercise-induced improvements.

In this study, exercise failed to significantly increase plasma GLP-1 levels in type 2 diabetic mice, which is consistent with a previous research finding (Eshghi et al., 2013). This phenomenon may be attributed to the alterations in GLP-1 secretion/function associated with T2DM mellitus (Andersen et al., 2018). However, our study revealed that fecal microbiota transplantation (FMT) from exercised type 2 diabetic mice to non-intervened diabetic mice resulted in significantly elevated GLP-1 levels. This demonstrates that the gut microbiota shaped by exercise can significantly elevate GLP-1 levels.

A previous study (Grasset et al., 2017) has revealed that gut dysbiosis contributes to GLP-1 resistance in obese and diabetic mice. Notably, diabetic mice exhibit increased relative abundances of Bacteroides, Burkholderia, and Clostridium. Importantly, the relative abundance of Bacteroides shows a negative correlation with ileal GLP-1 receptor (GLP-1R) and neuronal nitric oxide synthase (nNOS) mRNA expression. That’s why exercise failed to increase GLP-1 level in diabetic mice with gut microbiota dysbiosis, However, diabetic mice with FMT from mice undergoing exercise(mice with improved gut microbiota dysbiosis) increased GLP-1 level in diabetic mice.

This study demonstrated that exercise elevated Akkermansia and Prevotellaceae abundance and suppressed Escherichia_Shigella in type 2 diabetic mice. This beneficial effect may be attributed to short-chain fatty acids (SCFAs), including butyrate, acetate, and propionate, produced by exercise-enriched microbiota such as Akkermansia and Prevotellaceae. Specifically, Propionate upregulates GLP-1 expression in colonic tissues by activating FFAR2 (free fatty acid receptor 2) on enteroendocrine L cells, thereby potentiating GLP-1 secretion (Forbes et al., 2015).

In our study, *in vitro* experiments demonstrated that exercise-induced enhancement of proliferation and migration capacity in EPCs of type 2 diabetic mice was mediated by gut microbiota. Previous studies have established that both aerobic exercise and resistance exercise improve EPCs function in T2DM mice (Zhai et al., 2020). The current study further revealed that FMT from T2DM mice subjected to aerobic or resistance exercise into sedentary T2DM recipients similarly enhanced EPCs proliferation and migration. GLP-1, beyond its glucose-lowering effects, is known to promote vascular repair by augmenting EPC activity (Zhang et al., 2024). This may explain how gut microbiota ameliorates EPC proliferation and migration in type 2 diabetic mice, though the precise mechanisms remain to be elucidated.

### 4.2 Exercise-induced microbial remodeling

Our study revealed clear separation between exercise groups (AT, RT, AT+RT) and the sedentary control, with reduced overlap in microbial profiles post-exercise. In consistence with another study (Lambert et al., 2015), Prevotellaceae_UCG_001 group was enriched in exercise groups, aligning with prior studies linking these genera to produce short-chain fatty acids (SCFAs), such as propionate, which was found to reduce serum cholesterol levels and suppress hepatic lipid synthesis and reduce body weight, fasting blood glucose, fluid and food intake (Yu et al., 2019). The downregulation of Escherichia_Shigella (a pro-inflammatory pathobiont) in AT and RT groups in our study, alongside upregulation of Faecalibaculum and Ruminococcus (associated with short-chain fatty acid production), which were found to ameliorate hyperglycemia, insulin resistance, oxidative stress, and reduce liver lipid levels in T2D mice (Ye et al., 2024).

### 4.3 FMT recapitulates metabolic benefits with caveats

FMT from exercise-trained donors (AT+RT group) led to increased Akkermansia abundance, which is consistence with another study (Yu et al., 2019). Many studies have reported negative correlation of genus Akkermansia was reported to decline in patient with T2DM (Sedighi et al., 2017; Gao et al., 2018), assists in inhibition of α-glucosidase and reduction of postprandial hyperglycaemia (Everard et al., 2013), and reduce inflammation by inhibiting the expression of TNF-α and lipid oxidative damage in diabetic animals (Zhang et al., 2018). However, the concomitant decline in Prevotellaceae and Ruminococcaceae, accompanied by elevated Escherichia_Shigella in FMT and NS groups. Escherichia-Shigella was found to increase the most in fecal samples of the DM group, which was consistent with a previous study (Ejtahed et al., 2020), and was considered to be a kind of opportunistic pathogen for humans, which could produce various proinflammatory (Ramezani and Raj, 2014), components such as lipopolysaccharide (LPS) and peptidoglycans (PGN) and finally trigger development of diabetes (Chong et al., 2024). This unexpected result may reflect unintended consequences of pre-FMT antibiotic treatment (Girdhar et al., 2022), which likely disrupted native microbial networks. This underscores the importance of optimizing FMT protocols to preserve beneficial taxa.

### 4.4 Functional implications of microbial shifts

COG and KEGG annotations revealed exercise-induced downregulation of energy production and secondary metabolite pathways, possibly reflecting host energy redistribution during physical activity. Post-FMT, the NS group’s enhanced energy metabolism pathways suggest transplanted microbiota may optimize host energy harvesting efficiency, a double-edged sword that warrants further investigation in metabolic contexts.

## 5 Conclusion

Our study demonstrates that exercise intervention in diabetic mice significantly increased beneficial gut microbiota abundance and reduced blood glucose/body weight, though without altering GLP-1 levels. Strikingly, transplantation of exercise-modified gut microbiota not only elevated Akkermansia abundance but also markedly enhanced GLP-1 secretion in recipient mice, underscoring the pivotal role of gut microbiota in GLP-1 regulation. Furthermore, exercise-derived microbiota transplantation improved endothelial progenitor cell (EPC) proliferation and migration capacities in diabetic recipients.

## 6 Limitations and future work

While this study provides compelling evidence for the beneficial effects of exercise-modified microbiota on metabolic and vascular parameters, several limitations should be acknowledged, including the observed post-FMT increase in potentially harmful bacterial taxa and the need for more refined experimental designs to better isolate exercise-specific effects. Future investigations by our team will focus on elucidating whether the FMT-mediated improvements in GLP-1 secretion and EPC functionality are mechanistically dependent on exercise-induced microbial remodeling, as well as characterizing the precise molecular pathways underlying these therapeutic effects.

## Data Availability

The raw data supporting the conclusions of this article will be made available by the authors, without undue reservation.
