# Supplementary figures and images for: Exercise improves endothelial progenitor cell’s function in mice with Type 2 diabetes via gut microbiota modulation
Source: Front Cell Infect Microbiol. 2025 Aug 28;15:1606652. doi: 10.3389/fcimb.2025.1606652 (PMC12423053; doi:10.3389/fcimb.2025.1606652)

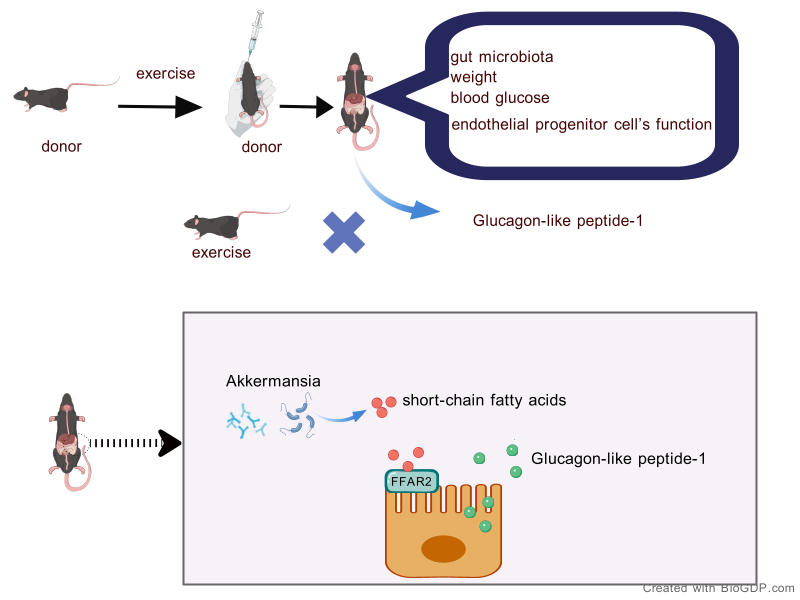

Supplement: Supplementary file 1 [file Image1.tiff]
