# Supplementary material for: Exercise improves endothelial progenitor cell’s function in mice with Type 2 diabetes via gut microbiota modulation
Source: Front Cell Infect Microbiol. 2025 Aug 28;15:1606652. doi: 10.3389/fcimb.2025.1606652 (PMC12423053; doi:10.3389/fcimb.2025.1606652)
Supplement: Supplementary file 2 [file Table1.docx]

| time | Control (n=6) | AT (n=6) | RT (n=6) | AT+RT (n=6) | P value a |
| --- | --- | --- | --- | --- | --- |
| 0W | 18.98±4.76 | 18.12±3.35 | 18.30±5.25 | 18.40±3.65 | 0.987 |
| 1W | 19.63±1.22 | 22.28±5.93 | 22.00±3.65 | 21.08±2.94 | 0.372 |
| 2W | 23.43±5.51 | 20.45±4.14 | 18.87±3.81 | 25.37±2.71 | 0.057 |
| 4W | 22.63±5.81 | 24.12±3.87 | 18.03±4.61 | 25.13±4.34 | 0.076 |
| 8W | 23.35±3.82 | 19.87±3.85 | 19.80±3.29 | 25.68±6.34 | 0.176 |

Multiple comparisons using Tukey's HSD test

| variable | Mean difference (95% CI) | P value |
| --- | --- | --- |
| 0W |  |  |
| AT vs. Control | -0.87 (-7.85, 6.12) | 0.985 |
| RT vs. Control | -0.68 (-7.67, 6.30) | 0.993 |
| AT+RT vs. Control | -0.58 (-7.57, 6.40) | 0.995 |
| RT vs. AT | 0.18 (-6.80, 7.17) | >0.999 |
| AT+RT vs. AT | 0.28 (-6.70, 7.27) | 0.999 |
| AT+RT vs. RT | 0.10 (-6.89, 7.09) | >0.999 |
| 1W |  |  |
| AT vs. Control | 2.65 (-3.54, 8.84) | 0.635 |
| RT vs. Control | 2.37 (-3.82, 8.56) | 0.711 |
| AT+RT vs. Control | 1.45 (-4.74, 7.64) | 0.912 |
| RT vs. AT | -0.28 (-6.47, 5.91) | 0.999 |
| AT+RT vs. AT | -1.20 (-7.39, 4.99) | 0.947 |
| AT+RT vs. RT | -0.92 (-7.11, 5.27) | 0.975 |
| 2W |  |  |
| AT vs. Control | -2.98 (-9.71, 3.74) | 0.609 |
| RT vs. Control | -4.57 (-11.29, 2.16) | 0.260 |
| AT+RT vs. Control | 1.93 (-4.79, 8.66) | 0.852 |
| RT vs. AT | -1.58 (-8.31, 5.14) | 0.911 |
| AT+RT vs. AT | 4.92 (-1.81, 11.64) | 0.205 |
| AT+RT vs. RT | 6.50 (-0.23, 13.23) | 0.061 |
| 4W |  |  |
| AT vs. Control | 1.48 (-6.13, 9.10) | 0.947 |
| RT vs. Control | -4.60 (-12.21, 3.01) | 0.354 |
| AT+RT vs. Control | 2.50 (-5.11, 10.11) | 0.795 |
| RT vs. AT | -6.08 (-13.70, 1.53) | 0.148 |
| AT+RT vs. AT | 1.02 (-6.60, 8.63) | 0.982 |
| AT+RT vs. RT | 7.10 (-0.51, 14.71) | 0.073 |
| 8W |  |  |
| AT vs. Control | -3.48 (-10.73, 3.76) | 0.546 |
| RT vs. Control | -3.55 (-10.80, 3.70) | 0.531 |
| AT+RT vs. Control | 2.33 (-4.91, 9.58) | 0.804 |
| RT vs. AT | -0.07 (-7.31, 7.18) | >0.999 |
| AT+RT vs. AT | 5.82 (-1.43, 13.06) | 0.145 |
| AT+RT vs. RT | 5.88 (-1.36, 13.13) | 0.138 |

Abbreviations: CI, confidence interval.
