# Supplementary material for: Exercise improves endothelial progenitor cell’s function in mice with Type 2 diabetes via gut microbiota modulation
Source: Front Cell Infect Microbiol. 2025 Aug 28;15:1606652. doi: 10.3389/fcimb.2025.1606652 (PMC12423053; doi:10.3389/fcimb.2025.1606652)
Supplement: Supplementary file 3 [file Table2.docx]

| time | Control (n=10) | PBS (n=10) | FMT (n=10) | P value a |
| --- | --- | --- | --- | --- |
| 0W | 19.86±0.96 | 19.47±1.06 | 19.82±1.25 | 0.686 |
| 1W | 20.27±1.03 | 20.20±0.86 | 20.12±1.00 | 0.942 |
| 2W | 21.84±0.88 | 21.77±0.87 | 21.52±0.69 | 0.659 |
| 4W | 21.60±1.09 | 23.96±1.01 | 23.81±1.89 | <0.001 |
| 8W | 29.34±0.69 | 28.39±1.16 | 20.12±0.94 | <0.001 |

Multiple comparisons using Tukey's HSD test

| variable | Mean difference (95% CI) | P value |
| --- | --- | --- |
| 0W |  |  |
| PBS vs. Control | -0.39 (-1.61, 0.83) | 0.710 |
| FMT vs. Control | -0.04 (-1.26, 1.18) | 0.996 |
| FMT vs. PBS | 0.35 (-0.87, 1.57) | 0.758 |
| 1W |  |  |
| PBS vs. Control | -0.07 (-1.14, 1.00) | 0.986 |
| FMT vs. Control | -0.15 (-1.22, 0.92) | 0.936 |
| FMT vs. PBS | -0.08 (-1.15, 0.99) | 0.981 |
| 2W |  |  |
| PBS vs. Control | -0.07 (-0.98, 0.84) | 0.980 |
| FMT vs. Control | -0.32 (-1.23, 0.59) | 0.660 |
| FMT vs. PBS | -0.25 (-1.16, 0.66) | 0.775 |
| 4W |  |  |
| PBS vs. Control | 2.36 (0.82, 3.90) | 0.002 |
| FMT vs. Control | 2.21 (0.67, 3.75) | 0.004 |
| FMT vs. PBS | -0.15 (-1.69, 1.39) | 0.968 |
| 8W |  |  |
| PBS vs. Control | -0.95 (-2.00, 0.10) | 0.083 |
| FMT vs. Control | -9.22 (-10.27, -8.17) | <0.001 |
| FMT vs. PBS | -8.27 (-9.32, -7.22) | <0.001 |

Abbreviations: CI, confidence interval.
