# Supplementary material for: Exercise improves endothelial progenitor cell’s function in mice with Type 2 diabetes via gut microbiota modulation
Source: Front Cell Infect Microbiol. 2025 Aug 28;15:1606652. doi: 10.3389/fcimb.2025.1606652 (PMC12423053; doi:10.3389/fcimb.2025.1606652)
Supplement: Supplementary file 4 [file Table3.docx]

| time | Control (n=5) | AT (n=5) | RT (n=5) | AT+RT (n=5) | P value a |
| --- | --- | --- | --- | --- | --- |
| 0W | 41.61±2.31 | 41.28±2.06 | 41.69±1.81 | 37.90±4.03 | 0.119 |
| 1W | 44.02±1.31 | 40.77±2.02 | 42.77±2.19 | 43.82±2.13 | 0.066 |
| 2W | 41.75±2.16 | 39.19±1.49 | 41.45±3.24 | 41.19±1.62 | 0.297 |
| 4W | 44.78±1.60 | 37.90±2.31 | 40.83±2.65 | 41.23±2.51 | 0.002 |
| 8W | 43.15±0.61 | 32.56±4.87 | 36.81±2.93 | 35.13±3.68 | 0.001 |

Multiple comparisons using Tukey's HSD test

| variable | Mean difference (95% CI) | P value |
| --- | --- | --- |
| 0W |  |  |
| AT vs. Control | -0.33 (-5.21, 4.55) | 0.997 |
| RT vs. Control | 0.08 (-4.80, 4.96) | >0.999 |
| AT+RT vs. Control | -3.71 (-8.59, 1.17) | 0.173 |
| RT vs. AT | 0.41 (-4.47, 5.29) | 0.995 |
| AT+RT vs. AT | -3.38 (-8.26, 1.50) | 0.236 |
| AT+RT vs. RT | -3.79 (-8.67, 1.09) | 0.159 |
| 1W |  |  |
| AT vs. Control | -3.24 (-6.76, 0.27) | 0.076 |
| RT vs. Control | -1.25 (-4.77, 2.27) | 0.743 |
| AT+RT vs. Control | -0.20 (-3.71, 3.32) | 0.998 |
| RT vs. AT | 2.00 (-1.52, 5.51) | 0.394 |
| AT+RT vs. AT | 3.05 (-0.47, 6.57) | 0.102 |
| AT+RT vs. RT | 1.05 (-2.47, 4.57) | 0.827 |
| 2W |  |  |
| AT vs. Control | -2.55 (-6.60, 1.49) | 0.307 |
| RT vs. Control | -0.30 (-4.35, 3.75) | 0.997 |
| AT+RT vs. Control | -0.56 (-4.60, 3.49) | 0.979 |
| RT vs. AT | 2.26 (-1.79, 6.30) | 0.409 |
| AT+RT vs. AT | 2.00 (-2.05, 6.05) | 0.510 |
| AT+RT vs. RT | -0.26 (-4.31, 3.79) | 0.998 |
| 4W |  |  |
| AT vs. Control | -6.88 (-11.04, -2.71) | 0.001 |
| RT vs. Control | -3.95 (-8.11, 0.22) | 0.066 |
| AT+RT vs. Control | -3.55 (-7.71, 0.62) | 0.110 |
| RT vs. AT | 2.93 (-1.24, 7.10) | 0.225 |
| AT+RT vs. AT | 3.33 (-0.83, 7.50) | 0.142 |
| AT+RT vs. RT | 0.40 (-3.76, 4.57) | 0.992 |
| 8W |  |  |
| AT vs. Control | -10.58 (-16.74, -4.43) | 0.001 |
| RT vs. Control | -6.33 (-12.49, -0.18) | 0.042 |
| AT+RT vs. Control | -8.02 (-14.17, -1.87) | 0.009 |
| RT vs. AT | 4.25 (-1.90, 10.40) | 0.237 |
| AT+RT vs. AT | 2.56 (-3.59, 8.72) | 0.640 |
| AT+RT vs. RT | -1.69 (-7.84, 4.47) | 0.861 |

Abbreviations: CI, confidence interval.
