# Supplementary material for: Exercise improves endothelial progenitor cell’s function in mice with Type 2 diabetes via gut microbiota modulation
Source: Front Cell Infect Microbiol. 2025 Aug 28;15:1606652. doi: 10.3389/fcimb.2025.1606652 (PMC12423053; doi:10.3389/fcimb.2025.1606652)
Supplement: Supplementary file 5 [file Table4.docx]

| time | Control (n=10) | PBS (n=10) | FMT (n=10) | P value a |
| --- | --- | --- | --- | --- |
| 0W | 49.31±1.00 | 49.54±1.30 | 49.48±1.02 | 0.892 |
| 1W | 49.66±0.83 | 49.97±1.21 | 49.98±1.15 | 0.753 |
| 2W | 50.29±0.89 | 50.63±1.04 | 50.50±1.05 | 0.746 |
| 4W | 49.58±0.73 | 52.55±1.70 | 51.94±1.34 | <0.001 |
| 8W | 53.70±1.53 | 52.34±2.25 | 49.01±0.86 | <0.001 |

Multiple comparisons using Tukey's HSD test

| variable | Mean difference (95% CI) | P value |
| --- | --- | --- |
| 0W |  |  |
| PBS vs. Control | 0.23 (-1.01, 1.47) | 0.890 |
| FMT vs. Control | 0.17 (-1.07, 1.41) | 0.938 |
| FMT vs. PBS | -0.06 (-1.30, 1.18) | 0.992 |
| 1W |  |  |
| PBS vs. Control | 0.31 (-0.88, 1.50) | 0.797 |
| FMT vs. Control | 0.32 (-0.87, 1.51) | 0.785 |
| FMT vs. PBS | 0.01 (-1.18, 1.20) | >0.999 |
| 2W |  |  |
| PBS vs. Control | 0.34 (-0.76, 1.44) | 0.728 |
| FMT vs. Control | 0.21 (-0.89, 1.31) | 0.885 |
| FMT vs. PBS | -0.13 (-1.23, 0.97) | 0.954 |
| 4W |  |  |
| PBS vs. Control | 2.97 (1.51, 4.43) | <0.001 |
| FMT vs. Control | 2.36 (0.90, 3.82) | 0.001 |
| FMT vs. PBS | -0.61 (-2.07, 0.85) | 0.562 |
| 8W |  |  |
| PBS vs. Control | -1.36 (-3.19, 0.47) | 0.175 |
| FMT vs. Control | -4.69 (-6.52, -2.86) | <0.001 |
| FMT vs. PBS | -3.33 (-5.16, -1.50) | <0.001 |

Abbreviations: CI, confidence interval.
