# Supplementary material for: Exercise improves endothelial progenitor cell’s function in mice with Type 2 diabetes via gut microbiota modulation
Source: Front Cell Infect Microbiol. 2025 Aug 28;15:1606652. doi: 10.3389/fcimb.2025.1606652 (PMC12423053; doi:10.3389/fcimb.2025.1606652)
Supplement: Supplementary file 6 [file Table5.docx]

| time | Control (n=5) | AT (n=5) | RT (n=5) | AT+RT (n=5) | P value a |
| --- | --- | --- | --- | --- | --- |
| 0W |  |  |  |  | 0.531 |
| 0W | 1.39±0.36 | 1.50±0.70 | 0.94±0.26 | 1.35±0.41 |  |
| 0W | 2 (40.0%) | 0 (0.0%) | 2 (40.0%) | 2 (40.0%) |  |
| 1W |  |  |  |  | 0.701 |
| 1W | 5.52±6.32 | 2.18±1.56 | 1.02±1.00 | 1.32±1.24 |  |
| 1W | 3 (60.0%) | 2 (40.0%) | 1 (20.0%) | 0 (0.0%) |  |
| 2W |  |  |  |  | 0.438 |
| 2W | 0.59±0.32 | 2.24±2.22 | 0.82±0.25 | 0.75±0.37 |  |
| 2W | 1 (20.0%) | 0 (0.0%) | 0 (0.0%) | 0 (0.0%) |  |
| 4W |  |  |  |  | 0.865 |
| 4W | 1.73±1.08 | 1.70±1.58 | 2.07±1.05 | 1.46±0.64 |  |
| 4W | 1 (20.0%) | 0 (0.0%) | 0 (0.0%) | 0 (0.0%) |  |
| 8W |  |  |  |  | 0.923 |
| 8W | 1.54±0.41 | 1.86±1.10 | 1.79±2.21 | 2.46±3.24 |  |
| 8W | 1 (20.0%) | 0 (0.0%) | 0 (0.0%) | 0 (0.0%) |  |

Multiple comparisons using Tukey's HSD test

| variable | Mean difference (95% CI) | P value |
| --- | --- | --- |
| 0W |  |  |
| AT vs. Control | 0.11 (-1.04, 1.27) | 0.991 |
| RT vs. Control | -0.46 (-1.75, 0.84) | 0.709 |
| AT+RT vs. Control | -0.04 (-1.33, 1.25) | >0.999 |
| RT vs. AT | -0.57 (-1.72, 0.59) | 0.473 |
| AT+RT vs. AT | -0.15 (-1.30, 1.01) | 0.978 |
| AT+RT vs. RT | 0.42 (-0.87, 1.71) | 0.759 |
| 1W |  |  |
| AT vs. Control | -3.34 (-9.83, 3.16) | 0.435 |
| RT vs. Control | -4.50 (-10.66, 1.66) | 0.179 |
| AT+RT vs. Control | -4.20 (-10.15, 1.75) | 0.200 |
| RT vs. AT | -1.17 (-6.60, 4.27) | 0.911 |
| AT+RT vs. AT | -0.86 (-6.06, 4.33) | 0.955 |
| AT+RT vs. RT | 0.30 (-4.47, 5.08) | 0.997 |
| 2W |  |  |
| AT vs. Control | 1.65 (-0.63, 3.92) | 0.201 |
| RT vs. Control | 0.22 (-2.05, 2.50) | 0.992 |
| AT+RT vs. Control | 0.15 (-2.12, 2.43) | 0.997 |
| RT vs. AT | -1.43 (-3.57, 0.72) | 0.263 |
| AT+RT vs. AT | -1.50 (-3.64, 0.65) | 0.227 |
| AT+RT vs. RT | -0.07 (-2.22, 2.07) | >0.999 |
| 4W |  |  |
| AT vs. Control | -0.04 (-2.24, 2.17) | >0.999 |
| RT vs. Control | 0.33 (-1.87, 2.54) | 0.971 |
| AT+RT vs. Control | -0.28 (-2.48, 1.93) | 0.983 |
| RT vs. AT | 0.37 (-1.71, 2.45) | 0.955 |
| AT+RT vs. AT | -0.24 (-2.32, 1.84) | 0.987 |
| AT+RT vs. RT | -0.61 (-2.69, 1.47) | 0.832 |
| 8W |  |  |
| AT vs. Control | 0.31 (-3.76, 4.39) | 0.996 |
| RT vs. Control | 0.25 (-3.83, 4.32) | 0.998 |
| AT+RT vs. Control | 0.91 (-3.16, 4.99) | 0.915 |
| RT vs. AT | -0.07 (-3.91, 3.78) | >0.999 |
| AT+RT vs. AT | 0.60 (-3.25, 4.44) | 0.969 |
| AT+RT vs. RT | 0.67 (-3.18, 4.51) | 0.958 |

Abbreviations: CI, confidence interval.
