# Supplementary material for: Exercise improves endothelial progenitor cell’s function in mice with Type 2 diabetes via gut microbiota modulation
Source: Front Cell Infect Microbiol. 2025 Aug 28;15:1606652. doi: 10.3389/fcimb.2025.1606652 (PMC12423053; doi:10.3389/fcimb.2025.1606652)
Supplement: Supplementary file 7 [file Table6.docx]

One-way ANOVA

| Characteristics | Control (n=10) | PBS (n=10) | FMT (n=10) | F | P value |
| --- | --- | --- | --- | --- | --- |
| value | 1.90±0.22 | 2.01±0.18 | 2.82±0.40 | 19.874 | <0.001 |

Data were mean±SD, unless otherwise specified.

Multiple comparisons using Tukey's HSD test

| Comparison | Mean difference (95% CI) | P value |
| --- | --- | --- |
| value |  |  |
| PBS vs. Control | 0.11 (-0.20, 0.43) | 0.645 |
| FMT vs. Control | 0.92 (0.60, 1.23) | <0.001 |
| FMT vs. PBS | 0.80 (0.49, 1.12) | <0.001 |

Abbreviations: CI, confidence interval.
